# Evaluation of Social Isolation, Loneliness, and Cardiovascular Disease Among Older Women in the US

**DOI:** 10.1001/jamanetworkopen.2021.46461

**Published:** 2022-02-02

**Authors:** Natalie M. Golaszewski, Andrea Z. LaCroix, Job G. Godino, Matthew A. Allison, JoAnn E. Manson, Jennifer J. King, Julie C. Weitlauf, Jennifer W. Bea, Lorena Garcia, Candyce H. Kroenke, Nazmus Saquib, Brad Cannell, Steve Nguyen, John Bellettiere

**Affiliations:** 1 Herbert Wertheim School of Public Health and Human Longevity Science, University of California, San Diego; 2 Laura Rodriguez Research Institute, Family Health Centers of San Diego, San Diego, California; 3 Center for Wireless and Population Health Systems, University of California, San Diego; 4 Exercise and Physical Activity Resource Center, University of California, San Diego; 5 Division of Preventive Medicine, Department of Family Medicine, University of California, San Diego; 6 Division of Preventive Medicine, Department of Medicine, Brigham and Women’s Hospital, Harvard Medical School, Boston, Massachusetts; 7 Department of Health Promotion Sciences, University of Arizona Cancer Center, Tucson, Arizona; 8 Veterans Affairs Palo Alto Health Care System, Stanford University School of Medicine, Stanford, California; 9 Department of Psychiatry and Behavioral Sciences, Stanford University School of Medicine, Stanford, California; 10 University of California Davis School of Medicine, Davis; 11 Division of Research, Kaiser Permanente Northern California, Oakland; 12 Department of Clinical Sciences, College of Medicine, Sulaiman Al Rajhi University, Al Bukayriyah, Saudi Arabia; 13 Department of Epidemiology, Human Genetics, and Environmental Sciences, University of Texas Health Science Center at Houston School of Public Health–Dallas Campus

## Abstract

**Importance:**

Social isolation and loneliness are increasing public health concerns and have been associated with increased risk of cardiovascular disease (CVD) among older adults.

**Objective:**

To examine the associations of social isolation and loneliness with incident CVD in a large cohort of postmenopausal women and whether social support moderated these associations.

**Design, Setting, and Participants:**

This prospective cohort study, conducted from March 2011 through March 2019, included community-living US women aged 65 to 99 years from the Women’s Health Initiative Extension Study II who had no history of myocardial infarction, stroke, or coronary heart disease.

**Exposures:**

Social isolation and loneliness were ascertained using validated questionnaires.

**Main Outcomes and Measures:**

The main outcome was major CVD, which was physician adjudicated using medical records and included coronary heart disease, stroke, and death from CVD. Continuous scores of social isolation and loneliness were analyzed. Hazard ratios (HRs) and 95% CIs for CVD were calculated for women with high social isolation and loneliness scores (midpoint of the upper half of the distribution) vs those with low scores (midpoint of the lower half of the distribution) using multivariable Cox proportional hazards regression models adjusting for age, race and ethnicity, educational level, and depression and then adding relevant health behavior and health status variables. Questionnaire-assessed social support was tested as a potential effect modifier.

**Results:**

Among 57 825 women (mean [SD] age, 79.0 [6.1] years; 89.1% White), 1599 major CVD events occurred over 186 762 person-years. The HR for the association of high vs low social isolation scores with CVD was 1.18 (95% CI, 1.13-1.23), and the HR for the association of high vs low loneliness scores with CVD was 1.14 (95% CI, 1.10-1.18). The HRs after additional adjustment for health behaviors and health status were 1.08 (95% CI, 1.03-1.12; 8.0% higher risk) for social isolation and 1.05 (95% CI, 1.01-1.09; 5.0% higher risk) for loneliness. Women with both high social isolation and high loneliness scores had a 13.0% to 27.0% higher risk of incident CVD than did women with low social isolation and low loneliness scores. Social support was not a significant effect modifier of the associations (social isolation × social support: *r*, –0.18; *P* = .86; loneliness × social support: *r*, 0.78; *P* = .48).

**Conclusions and Relevance:**

In this cohort study, social isolation and loneliness were independently associated with modestly higher risk of CVD among postmenopausal women in the US, and women with both social isolation and loneliness had greater CVD risk than did those with either exposure alone. The findings suggest that these prevalent psychosocial processes merit increased attention for prevention of CVD in older women, particularly in the era of COVID-19.

## Key Points

**Question:**

Are there associations between social isolation, loneliness, and cardiovascular disease (CVD) among older women?

**Findings:**

In this cohort study of 57 825 older women in the US, social isolation and loneliness were associated with an 8.0% and 5.0% higher risk for incident CVD, respectively, after adjusting for health behaviors and outcomes. Women with greater social isolation and greater loneliness had a 13.0% to 27.0% higher risk of incident CVD compared with women with less social isolation and less loneliness.

**Meaning:**

In this study, social isolation and loneliness were associated with increased risk of incident CVD among older women in the US, suggesting that interventions to reduce social isolation and loneliness in this population are warranted.

## Teaser

This cohort study uses data from the Women’s Health Initiative to examine associations of social isolation and loneliness with incident cardiovascular disease among postmenopausal women in the US.

## Introduction

In the US, cardiovascular disease (CVD) is the leading cause of death in women^1^ and accounts for approximately 1 in every 5 deaths among women.^2^ Although there have been marked decreases in CVD mortality among both men and women, data suggest slowing of these decreases in CVD incidence and mortality among women.^3^ Social isolation and loneliness are prevalent psychosocial processes among adults^4^ and have been shown to be associated with increased risk of CVD among older adults.^5^ More than one-fourth of adults aged 65 or older are socially isolated, and one-third of adults aged 45 or older report being lonely.^6^ Of note, there is increasing evidence that both social isolation and loneliness are associated with CVD risk factors such as increased blood pressure,^7^ higher cholesterol levels, obesity, smoking,^8,9^ physical inactivity,^10,11^ and poor diet quality.^12^ Whereas social isolation is the objective measure of social interactions and relationships, loneliness is the subjective feeling of being socially isolated.^6,13^ The experiences of social isolation and feelings of loneliness have been shown to be mildly correlated^14^; however, they are distinct constructs, and research has shown that social isolation and loneliness are associated with different adverse outcomes of health and well-being.^14,15,16,17^

Older adults are particularly at risk for social isolation and loneliness as they encounter significant life changes, such as retirement, deaths of family members and friends, and decreasing activities of daily living. These life changes contribute to decreasing social network size, which influences already limited interactions among social contacts.^4^ Some evidence suggests that older women tend to experience more social isolation compared with men because they are more likely to be widowed and live alone.^18^ Older women who reported stronger feelings of loneliness were at an increased risk of incidence of coronary heart disease (CHD) compared with men.^19^ Some women are more connected to communities and family, which can lead to higher levels of social support, but they may still feel lonely or experience social isolation.^18^ The purpose of this study was to examine the associations of both social isolation and loneliness with incident CVD among a large sample of postmenopausal US women and assess the potential modifying role of social support. We hypothesized that there would be associations between social isolation, loneliness, and risk of incident CVD and that fewer associations with risk of CVD would be found among women with greater social support.

## Methods

### Participants

This cohort study, conducted from March 2011 through March 2019, included surviving women aged 65 to 99 years from the original Women’s Health Initiative (WHI) study (design, recruitment, and implementation described elsewhere^20,21^) who were enrolled in the WHI Extension Study II in 2010 (n = 93 500). From 2011 to 2012, questionnaires assessing several components of social isolation were sent to 81 487 women, and 73 709 (90.5%) provided complete data to measure social isolation (Figure). From 2014 to 2015, women were sent an additional questionnaire assessing loneliness and social support. A total of 61 161 women (75.1%) provided complete data on both measures; 3336 women (5.5%) with a history of myocardial infarction, stroke, and/or CHD were excluded from the sample. The remaining 57 825 women (94.5%) were followed up from the time of questionnaire completion through March 31, 2019, or their first reported major CVD event (Figure). The protocol for this study was approved by the Fred Hutchinson Cancer Research Center institutional review board, and all women provided informed consent either in writing or by telephone using an institutional review board–approved script. This study followed the Strengthening the Reporting of Observational Studies in Epidemiology (STROBE) reporting guideline.

**Figure.**
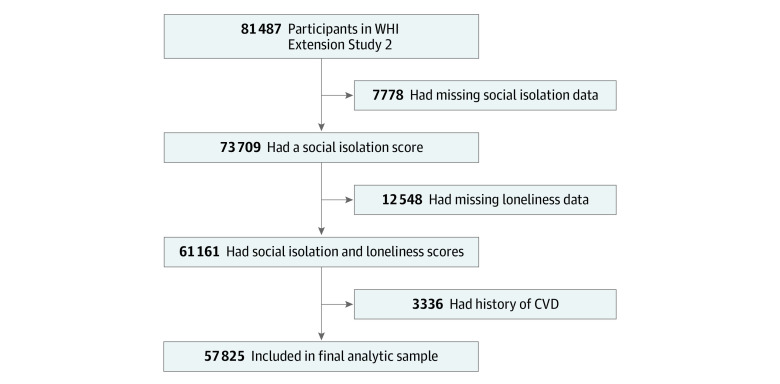
Flowchart for the Final Analytic Sample CVD indicates cardiovascular disease; WHI, Women’s Health Initiative.

### Measures

#### Social Isolation and Loneliness

A social isolation index score was modeled after a similar score^22^ and computed from marital status, living situation, and frequency of social activities. Participants responded “yes” (1) or “no” (0) to the following questions: “Are you currently married or in an intimate relationship with at least 1 person?” and “Do you live alone?” Participants also responded on a 4-point Likert scale (“rarely or never” [1], “once a month” [2], “several times a month” [3], or “at least once a week” [4]) to the question “How often, if at all, do you do any of the following activities? (a) Meet with family or friends who do not live with you; (b) Communicate with family or friends by phone or email; (c) Go to a church or other religious center; (d) Go to a cultural event such as a movie, concert, play, or lecture; (e) Eat out of the house; and (f) Go shopping.” Each activity was dichotomized such that a response of “rarely or never” was coded as 1 and all other responses were coded as 0. With use of methods previously implemented among older adults,^22^ the social isolation index score (range, 0-8) was calculated as the sum, with higher scores indicating greater social isolation.

Loneliness was measured by the 3-item UCLA Loneliness Scale.^23^ This scale reflects feelings of isolation, lack of companionship, and being left out. For example, 1 item used was “How often do you feel you lack companionship?” Response options (on a 3-point Likert scale) were “hardly ever or never” (1), “some of the time” (2), and “often” (3). Scores were summed and divided by 3 to provide a mean loneliness score (1-3), with higher scores indicating greater loneliness. This scale has shown good internal consistency, discriminant validity, and convergent validity among older adults.^23^

#### Social Support

Social support was assessed using 9 items from the 19-item Medical Outcomes Study Social Support Survey.^24^ Participants were given the following prompt: “People sometimes look to others for help, friendship, or other types of support. Next are some questions about the support that you have. How often is each of the following kinds of support available to you if you need it?” For example, 1 item included “Someone you can count on to listen to you when you need to talk,” with response options on a 5-point Likert scale ranging from “none of the time” (1) to “all of the time” (5). A total score (range, 9-45) was calculated, with higher scores reflecting greater social support. Previous WHI studies have used this social support scale with postmenopausal women.^25,26,27^

#### Ascertainment of CVD

Annual medical updates with information related to new CVD events were collected from each participant, and the first reported occurrence of each event was adjudicated by study physicians (including M.A.A.) through a medical record review of the corresponding hospitalizations. Major CVD included the first reported occurrence of CHD (clinical myocardial infarction, definite silent myocardial infarction, or death attributable to definite CHD or possible CHD), stroke, or death attributable to any CVD. Detailed descriptions of the WHI CVD adjudication process were published previously.^28,29^ Interrater agreement for CVD ascertainment ranged from κ = 0.67-0.94.^28^

#### Covariates

Factors (including age and educational level) that may confound associations of social isolation and loneliness with CVD were selected based on previous literature.^30,31,32^ Self-reported race and ethnicity were included because previous research showed that associations between social isolation,^33^ loneliness,^34^ and CVD^35^ differed by race and ethnicity. Differences likely reflect structural racism and not biological differences.^36^ Physical functioning was assessed using the RAND 36-Item Short Form Survey 10-item physical functioning subscale, with scores ranging from 0 to 100 and higher scores indicating greater physical functioning.^37^ Self-reported alcohol consumption (nondrinker, <1 drink per week, ≥1 drink per week, or every day) and smoking status (never, former, or current) were assessed. Physical activity was assessed through energy expenditure from mild exercises (eg, walking or golf) in metabolic equivalent hours per week. Diet quality was assessed using the validated Healthy Eating Index,^38^ scored from 0 to 100 with higher scores indicating closer conformance with dietary guidance. Depression (yes or no) and diabetes (yes or no) were assessed by questionnaire along with history or current use of hypertension medication (yes or no) and/or hyperlipidemia medication (yes or no).

### Statistical Analysis

Participant characteristics were described for women with social isolation scores higher or lower than the median of 1 and loneliness scores higher or lower than the median of 0.33. Restricted cubic splines tested the assumption of a linear association of social isolation and loneliness with risk for CVD using 3, 4, and 5 knots. We inspected the dose-response trajectory for evidence of nonlinearity and the *P* value from a Wald test of the nonlinear component. Hazard ratios (HRs) and 95% CIs were estimated from sequentially adjusted Cox proportional hazards regression models as follows: model 1 was adjusted for age, race and ethnicity, educational level, and history of depression; model 2 was additionally adjusted for social isolation or loneliness to test for independent associations of each exposure variable; model 3 added health behaviors (smoking, alcohol consumption, and physical activity); and model 4 added health status variables (history of diabetes, hypertension medication use, hyperlipidemia medication use, general health, and physical functioning). Models 2 through 4 were then fit with an interaction term (social isolation × loneliness) to allow more flexibility when estimating the CVD risk ratio comparing high social isolation and high loneliness scores vs low social isolation and low loneliness scores. Ranges and distributions of the social isolation and loneliness measures were markedly different; thus, HRs are presented comparing women with high scores (midpoint of the upper half of the distribution) with women with low scores (midpoint of the lower half of the distribution) for each measure. Social support was tested as an effect modifier in model 4. Given the small percentage (2.4%) of missing covariate data, all nonmissing data points were used in the analyses. To address the assumption that women who died of causes other than major CVD were at risk of developing major CVD, we performed a sensitivity analysis using the Fine and Gray approach to competing risks implemented in the *cmprsk* package in R, version 1.4.1717 (R Project for Statistical Computing).^39^

Descriptive analyses were performed using SPSS, version 27 (IBM).^40^ All other analyses were conducted in R, version 1.4.1717.^41^ The significance level was set at 2-tailed *P* < .05.

## Results

Among the 57 825 women with complete data, the mean (SD) age was 79.0 [6.1] years; 0.3% identified as American Indian or Alaskan Native, 2.2% as Asian or Pacific Islander, 5.0% as Black or African American, 2.4% as Hispanic or Latina, 89.1% as non-Hispanic White, and 0.9% as other (the “other” category was not further disaggregated in the questionnaire). The greater proportion of women (51.3%) did not have a college degree (Table 1). Compared with the women with complete responses, the 20 236 women who were missing responses for either the social isolation or loneliness scales (excluding prevalent CVD at baseline) were older (mean [SD] age, 81.4 [6.4] years vs 79.0 [6.1] years), were more likely to report fair or poorer self-rated health (17.4% vs 6.1%), and had lower scores on the RAND 36-Item Short Form Survey 10-item physical functioning subscale (mean [SD], 57.9 [29.3] vs 72.0 [24.4]). However, scores on the loneliness scale among the 7778 excluded women who provided answers were higher compared with those of women included in the study (mean [SD], 1.29 [0.46] vs 1.24 [0.42]), and levels of social isolation among the 14 953 women who were excluded but provided answers were also higher (mean [SD] score, 2.00 [1.45] vs 1.45 [1.19]).

**Table 1.** Baseline Characteristics and Factors Associated With Cardiovascular Disease Risk Among Women’s Health Initiative Participants by Social Isolation and Loneliness Status^a^

Characteristic	Participants^b^				
	Total (N = 57 825)	Social isolation^c^		Loneliness^d^	
		Low score (n = 32 695)	High score (n = 25 130)	Low score (n = 37 763)	High score (n = 20 062)
Age, mean (SD), y	79.0 (6.1)	78.3 (5.7)	80.0 (6.4)	78.5 (5.9)	79.9 (6.3)
Race and ethnicity					
American Indian or Alaskan Native	175 (0.3)	90 (0.3)	85 (0.3)	110 (0.3)	65 (0.3)
Asian or Pacific Islander	1258 (2.2)	632 (1.9)	626 (2.5)	925 (2.5)	333 (1.7)
Black or African American	2893 (5.0)	1315 (4.0)	1578 (6.3)	1815 (4.8)	1078 (5.4)
Hispanic or Latina	1372 (2.4)	745 (2.3)	627 (2.5)	886 (2.4)	486 (2.4)
Non-Hispanic White	51 500 (89.1)	29 578 (90.6)	21 922 (87.4)	33 640 (89.2)	17 860 (89.2)
Other^e^	521 (0.9)	279 (0.9)	242 (1.0)	324 (0.9)	197 (1.0)
Highest educational level					
High school or less	9414 (16.4)	4935 (15.2)	4479 (17.9)	6001 (16.0)	3413 (17.1)
Some college or vocational training	20 276 (35.2)	11 251 (34.6)	9025 (36.1)	13 082 (34.8)	7194 (36.1)
College degree or higher	27 851 (48.4)	16 358 (50.3)	11 493 (46.0)	18 511 (49.2)	9340 (46.8)
Health behavior or status					
Current smoker	1150 (2.0)	387 (1.2)	763 (3.1)	625 (1.7)	525 (2.7)
Alcohol intake in past 3 mo					
Nondrinker	16 557 (28.6)	8304 (25.4)	8253 (32.8)	10 473 (27.7)	6084 (30.3)
<1 Drink/wk	18 532 (32.0)	10 460 (32.0)	8072 (32.1)	11 831 (31.3)	6701 (33.4)
≥1 Drink/wk	16 878 (29.2)	10 532 (32.2)	6346 (25.3)	11 558 (30.6)	5320 (26.5)
Every day	5226 (9.0)	3077 (9.4)	2149 (8.6)	3529 (9.3)	1697 (8.5)
Unknown	632 (1.1)	322 (1.0)	310 (1.2)	372 (1.0)	260 (1.3)
Self-rated health					
Excellent or very good	34 571 (60.6)	21 083 (65.3)	13 488 (54.5)	25 033 (67.1)	9538 (48.4)
Good	18 890 (33.1)	9868 (30.6)	9022 (36.5)	10 783 (28.9)	8107 (41.1)
Fair or poor	3541 (6.2)	1317 (4.1)	2224 (9.0)	1464 (3.9)	2077 (10.5)
Social support score, mean (SD)^f^	38.1 (7.4)	39.7 (6.4)	36.0 (8.2)	40.0 (6.2)	34.4 (8.1)
History of depression	3491 (6.0)	1530 (4.7)	1961 (7.8)	1087 (2.9)	2404 (12.0)
History of diabetes	8687 (15.0)	4530 (13.9)	4157 (16.5)	5266 (13.9)	3421 (17.1)
Hypertension medication use	34 107 (59.0)	18 723 (57.3)	15 384 (61.2)	21 752 (57.6)	12 355 (61.6)
Hyperlipidemia medication use	25 526 (44.1)	14 577 (44.6)	10 949 (43.6)	16510 (43.7)	9016 (44.9)
Healthy Eating Index score, mean (SD)	67.81 (10.03)	68.33 (9.71)	67.12 (10.41)	68.13 (9.93)	67.20 (10.20)
MET-h/wk, mean (SD)	1.76 (3.57)	1.91 (3.64)	1.57 (3.47)	1.90 (3.72)	1.51 (3.27)
Physical functioning score, mean (SD)^g^	72.0 (24.4)	75.4 (22.1)	67.6 (26.5)	75.5 (22.9)	65.5 (25.9)
Loneliness score, mean (SD)^d^	1.2 (0.4)	1.2 (0.4)	1.3 (0.5)	1.0 (0.0)	1.7 (0.42)
Social isolation score, mean (SD)^c^	1.5 (1.2)	0.6 (0.5)	2.6 (0.8)	1.3 (1.1)	1.8 (1.3)

Abbreviation: MET, metabolic equivalent.

^a^ Baseline characteristics were collected from 2011 to 2015.

^b^ Data are presented as the number (percentage) of participants unless otherwise indicated.

^c^ Adapted social isolation index score^22^; scores ranged from 0 to 8. A low score was below the median of 1 and a high score, above the median of 1.

^d^ UCLA Loneliness Scale^23^; scores ranged from 1 to 3. A low score was below the median of 0.33 and a high score, above the median of 0.33.

^e^ The category for Other was not further disaggregated in the study questionnaire.

^f^ Medical Outcomes Study Social Support Survey.^24^

^g^ RAND 36-Item Short Form Survey Instrument 10-item physical functioning subscale.^37^

Higher percentages of participants with above-median social isolation and loneliness had depression and diabetes, and these women were more likely to be smokers, consume less than 1 drink per week, and report poorer self-rated health, less social support, lower physical activity, and lower physical functioning. Social isolation was correlated with loneliness (*r*, 0.21; *P* < .001).

A total of 1599 major CVD events occurred in the study population over 186 762 person-years. Unadjusted rates of major CVD events were higher among women with above-median social isolation (11.9 per 1000 person-years) than among those with below-median social isolation (6.1 per 1000 person-years) based on the median split. The rate of CVD events among women with above-median loneliness was 11.5 per 1000 person-years and among women with below-median loneliness was 7.4 per 1000 person-years.

Restricted cubic spline analyses (eAppendix in the Supplement) indicated that the correlation between social isolation and incident CVD was linear (3 knots: nonlinear *P* = .58; 4 and 5 knots: nonlinear *P* = .06), with plots exhibiting a pattern indicative of overfitting. There was a linear correlation between loneliness and incident CVD (3 knots: nonlinear *P* = .30; 4 knots: nonlinear *P* = .41; and 5 knots: nonlinear *P* = .29). We used social isolation and loneliness in continuous functional form in linear models.

After adjustment for the covariates in model 1, the HR for the association of high vs low social isolation scores with incident CVD was 1.18 (95% CI, 1.13-1.23; 18.0% higher risk); the HR decreased to 1.16 (95% CI, 1.11-1.20; 16.0% higher risk) in model 2, to 1.13 (95% CI, 1.09-1.18; 13.0% higher risk) in model 3, and to 1.08 (95% CI, 1.03-1.12; 8.0% higher risk) in model 4 (Table 2). For loneliness, the HRs for the association of high vs low loneliness scores with incident CVD were 1.14 (95% CI, 1.10-1.18; 14.0% higher risk) in model 1, 1.11 (95% CI, 1.07-1.15; 11.0% higher risk) in model 2, 1.10 (95% CI, 1.06-1.14; 10.0% higher risk) in model 3, and 1.05 (95% CI, 1.01-1.09; 5.0% higher risk) in model 4 (Table 2). The HRs for the association of both high social isolation scores and high loneliness scores vs both low social isolation scores and low loneliness scores with incident CVD were 1.27 (95% CI, 1.21-1.36; 27.0% higher risk) in model 2, 1.24 (95% CI, 1.17-1.32; 24.0% higher risk) in model 3, and 1.13 (95% CI, 1.06-1.20; 13.0% higher risk) in model 4. In sensitivity analyses that accounted for the competing risk of death from nonmajor CVD, the HRs for the associations of social isolation and loneliness with major CVD did not appreciably change (eTable in the Supplement).

**Table 2.** Hazard Ratios for Associations of Social Isolation and Loneliness With Incident Major Cardiovascular Disease Among 57 825 Older Women in the Women’s Health Initiative

Model	Hazard ratio (95% CI)^a^		
	Social isolation^b^	Loneliness^c^	Social isolation and loneliness
1^d^	1.18 (1.13-1.23)	1.14 (1.10-1.18)	NA
2^e^	1.16 (1.11-1.20)	1.11 (1.07-1.15)	1.27 (1.21-1.36)
3^f^	1.13 (1.09-1.18)	1.10 (1.06-1.14)	1.24 (1.17-1.32)
4^g^	1.08 (1.03-1.12)	1.05 (1.01-1.09)	1.13 (1.06-1.20)

Abbreviation: NA, not applicable.

^a^ Hazard ratios compare women with high (midpoint of the upper half of the distribution) vs low (midpoint of the lower half of the distribution) levels of each measure.

^b^ The median for social isolation was 1.

^c^ The median for loneliness was 0.33.

^d^ Model 1 was adjusted for age, race and ethnicity, educational level, and depression.

^e^ Model 2 was adjusted for model 1 criteria plus loneliness, social isolation, or both depending on the exposure.

^f^ Model 3 was adjusted for model 2 criteria plus smoking status, frequency of alcohol consumption, history of depression, physical activity, and diet.

^g^ Model 4 was adjusted for model 3 criteria plus history of diabetes, use of hypertension medication, use of hyperlipidemia medication, overall health, and physical functioning.

### Social Support as an Effect Modifier

Social support (mean [SD] score, 38.1 [7.4]) was inversely correlated with both social isolation (*r*, −0.31; *P* < .001) and loneliness (*r*, −0.39, *P* < .001). It was not a significant effect modifier for the associations of incident CVD with social isolation (social isolation × social support: *r*, –0.18; *P* = .86; loneliness × social support: *r*, 0.78; *P* = .48).

## Discussion

In this cohort study of postmenopausal women in the US, social isolation and loneliness were associated with a higher risk of incident CVD. After adjusting for a wide range of health behaviors and outcomes, some of which may be associated with social isolation and loneliness, higher social isolation and loneliness scores remained associated with an 8.0% and 5.0% higher risk for incident CVD, respectively. In the same fully adjusted model, women who had high levels of both social isolation and loneliness had a 13.0% higher risk for CVD compared with women with low levels of social isolation and loneliness. The mechanisms through which social isolation and loneliness are associated with incident CVD may partially involve health behaviors and changing health status,^42^ although in this study, the results suggest that the associations were not fully explained by these factors. Whereas we expected social support to have a moderating role in these associations, the results indicated that it did not.

Our findings contribute to the increasing literature focused on associations of social isolation and loneliness with risk of incident CVD. A systematic review and meta-analysis^43^ reported that across 23 studies, when comparing high vs low loneliness or social isolation, the mean relative risk of new CHD was 29% and the mean relative risk of stroke incidence was 32% among adults aged 18 years or older. The authors did not identify any significant differences by gender. The observed differences between that study and our study are partially owed to the heterogeneous social isolation and loneliness measures used in the studies reviewed.^43^ This required the researchers to dichotomize the measures into high vs low loneliness and social isolation, which may have increased the reported effect sizes.^44^ In our study, we estimated risk across the full distribution of social isolation and loneliness by including it in our models in continuous functional form. The wide age range of individuals included in the aforementioned systematic review and meta-analysis^43^ means that some risk ratios were estimated comparing younger participants (who were therefore at lower risk for CVD) with older participants, possibly leading to differences in results. The findings from the current study are consistent with those of a study^45^ among older adults that showed that after adjusting for potential confounders, social isolation and loneliness were associated with an 8% and 3% increased risk of incident CVD, respectively.

Our study’s findings are consistent with those of previous work that showed that social isolation and loneliness are distinct constructs based on their relatively low correlation with each other and their independent associations with incident CVD.^14^ Research has shown that social isolation and loneliness are associated with chronic stress^4,5^ and often together with health outcomes, such as CVD. Over time, the experiences of social isolation and feelings of loneliness may affect cardiovascular health by stimulating neuroendocrine dysregulation,^46^ disturbing autonomic function and increasing systolic blood pressure control,^47,48^ potentially sparking inflammatory responses,^49,50^ and increasing long-term allostatic load.^51^ A study^46^ suggested that social isolation and loneliness activate the hypothalamic-pituitary-adrenal axis and the sympathetic nervous system, leading to enhanced inflammation and oxidative stress, which may then lead to atherosclerosis development and systolic blood pressure elevation. Atherosclerosis and higher systolic blood pressure^7,42^ are associated with increased total peripheral resistance,^52^ which leads to arterial stiffening and increased systolic blood pressure.^52^

Longitudinal studies showed that individuals who experienced social isolation and loneliness had more behavioral factors, including smoking,^8,9^ alcohol use,^53^ physical inactivity,^10,11^ and poor diet quality,^12^ that may further be associated with the progression of CVD. Our findings showed that postmenopausal women with high social isolation and loneliness scores vs low social isolation and loneliness scores had a greater likelihood of reporting a history of diabetes and depression and that a greater percentage reported currently smoking, consuming less than 1 drink per week, and having poorer overall health, less social support, and lower levels of physical activity and physical functioning. After adjusting for health behaviors and outcomes, the HRs for the associations of social isolation and loneliness with CVD were lower. Associations between these experiences and CVD may partly be explained by health behaviors and outcomes. The extent to which social isolation and/or loneliness are associated with risks of poor health behaviors or whether having poor health is associated with an increased risk of social isolation and loneliness is important to understand. Our study lacked sufficient repeated measures of these factors to evaluate the potential bidirectionality of the associations. Future research should explore these patterns to understand the dynamics of social isolation, loneliness, health behaviors, health outcomes, and incident CVD.

Contrary to our hypothesis, social support did not modify the association of social isolation or loneliness with CVD. Social support has been identified to be associated with decreased levels of health stressors^54^ and, specifically, with reductions in cardiovascular reactivity to stress.^55,56,57^ In this study, women reported a mean social support score of approximately 38 on a scale with a theoretical range of 9 to 45, suggesting scores in the higher range. The Medical Outcomes Study Social Support Survey provides a composite score of emotional and informational support, tangible support, positive social interaction, and affection.^24^ It is possible that too few women had lower levels of social support to observe a modifying effect. Further research is needed on identifying forms of social support that attenuate the distress associated with social isolation and loneliness. Findings from such research may help identify tangible ways for individuals who are socially isolated or feeling lonely to stay socially connected. For example, the National Institute on Aging recommends scheduling times each day to keep in touch with family, friends, and neighbors.^58^ In addition, joining group-based exercise to stay physically active may also provide a sense a belonging.^59^

### Limitations

This study has limitations. The necessary exclusion of a large number of women with missing data on either loneliness or social support, who were on average older and had poorer self-rated health status and lower physical functioning levels but modestly higher levels of social isolation, could have led to underestimation of the HR for the association between social isolation and CVD risk. This study also could have been strengthened by a shorter time lag between the exposure variable measures; there were approximately 4 years between the measurement of social isolation and loneliness. As individuals age, their social networks decrease at a faster rate, and having proximal measures—and even better, repeated measures—would reduce misclassification and address more nuanced research questions about how changes in social isolation and loneliness are associated with CVD. In addition, we did not have a measure of body mass index for the entire sample; thus, we could not adjust for body mass index. However, we were able to adjust for behaviors such as physical activity and healthy eating.

## Conclusions

In this cohort study, social isolation and loneliness were independently associated with an 11.0% to 16.0% higher risk of CVD among a sample of older women in the US. In addition, higher levels of social isolation and loneliness were associated with a 13.0% to 27.0% higher risk of CVD. Even after adjusting for health behaviors and health outcomes, we observed a 5.0% and 8.0% higher risk of incident CVD among women with social isolation and loneliness, respectively, and a 13.0% higher risk among women with both high social isolation and high loneliness scores. The findings suggest that further research is needed to evaluate the effectiveness of various interventions to reduce social isolation and loneliness and their potential effects on CVD risk as well as overall quality of life. At present, particularly acknowledging the widespread consequences of the COVID-19 pandemic for these psychosocial conditions, the study’s findings support the potential value of measuring social isolation and loneliness in primary care and referring older women to mental health or community resources to improve social connectedness and reduce feelings of loneliness.

## Notes

Supplement. **eTable.** Hazard Ratios for the Associations of Social Isolation and Loneliness Accounting for Competing Risk of Nonmajor CVD Death (N = 57 825) **eAppendix.** Cubic Spline Analyses With 3, 4, and 5 Knots to Model Social Isolation (Panel A) and Loneliness (Panel B) in Relation to Risk for CVD Among Older Women (N = 57 825)
