# Supplementary material for: Evaluation of Social Isolation, Loneliness, and Cardiovascular Disease Among Older Women in the US
Source: JAMA Netw Open. 2022 Feb 2;5(2):e2146461. doi: 10.1001/jamanetworkopen.2021.46461 (PMC8811637; doi:10.1001/jamanetworkopen.2021.46461)
Supplement: Supplement. — eTable. Hazard Ratios for the Associations of Social Isolation and Loneliness Accounting for Competing Risk of Nonmajor CVD Death (N = 57 825) eAppendix. Cubic Spline Analyses With 3, 4, and 5 Knots to Model Social Isolation (Panel A) and Loneliness (Panel B) in Relation to Risk for CVD Among Older Women (N = 57 825) [file jamanetwopen-e2146461-s001.pdf]

Supplementary Online Content

Golaszewski NM, LaCroix AZ, Godino JG, et al. Evaluation of social isolation, loneliness, and cardiovascular disease among older women in the US. *JAMA Netw Open*. 2022;5(2):e2146461. doi:10.1001/jamanetworkopen.2021.46461

eTable. Hazard Ratios for the Associations of Social Isolation and Loneliness Accounting for Competing Risk of Nonmajor CVD Death (N = 57 825)
eAppendix. Cubic Spline Analyses With 3, 4, and 5 Knots to Model Social Isolation (Panel A) and Loneliness (Panel B) in Relation to Risk for CVD Among Older Women (N = 57 825)

This supplementary material has been provided by the authors to give readers additional information about their work.

© 2022 Golaszewski NM et al. *JAMA Network Open*.

eTable 1. Hazard Ratios for the Associations of Social Isolation and Loneliness accounting for Competing Risk of Non-major CVD death (n=57,825)

	**Social Isolation** [e]		**Loneliness**[f]	
	HR (95% CI)		HR (95% CI)	
	Main Analysis	Competing Risk	Main Analysis	Competing Risk
Model 1[a]	1.18 (1.13 - 1.23)	1.17 (1.12-1.21)	1.14 (1.10 - 1.18)	1.12 (1.08-1.16)
Model 2[b]	1.16 (1.11 - 1.20)	1.14 (1.1-1.19)	1.11 (1.07 - 1.15)	1.10 (1.06-1.14)
Model 3[c]	1.13 (1.09 - 1.18)	1.12 (1.07-1.17)	1.10 (1.06 - 1.14)	1.08 (1.05-1.12)
Model 4[d]	1.08 (1.03 - 1.12)	1.07 (1.02-1.11)	1.05 (1.01 - 1.09)	1.04 (1.00-1.08)
a Model 1 was adjusted for age, race/ethnicity, education, and depression.				
b Model 2 was adjusted for Model 1 plus either loneliness or social isolation depending on the exposure.				
c Model 3 was adjusted for Model 2 plus smoking status, frequency of alcohol consumption, history of depression, physical activity, and diet.				
d Model 4 was adjusted for Model 3 plus history of diabetes, hypertension medication use, hyperlipidemia medication use, overall health, and physical function.<br>e Median for social isolation is 1.<br>f Median for loneliness is 0.33.<br>* CVD: cardiovascular disease; HR: hazard ratio; CI: confidence interval<br>** Main analysis appears in Table 2 of the manuscript.				

© 2022 Golaszewski NM et al. *JAMA Network Open*.

eAppendix. Cubic spline analyses with 3, 4, and 5 knots to model social isolation (Panel A) and loneliness (Panel B) in relation to risk for CVD among older women (n=57,825)

Panel A

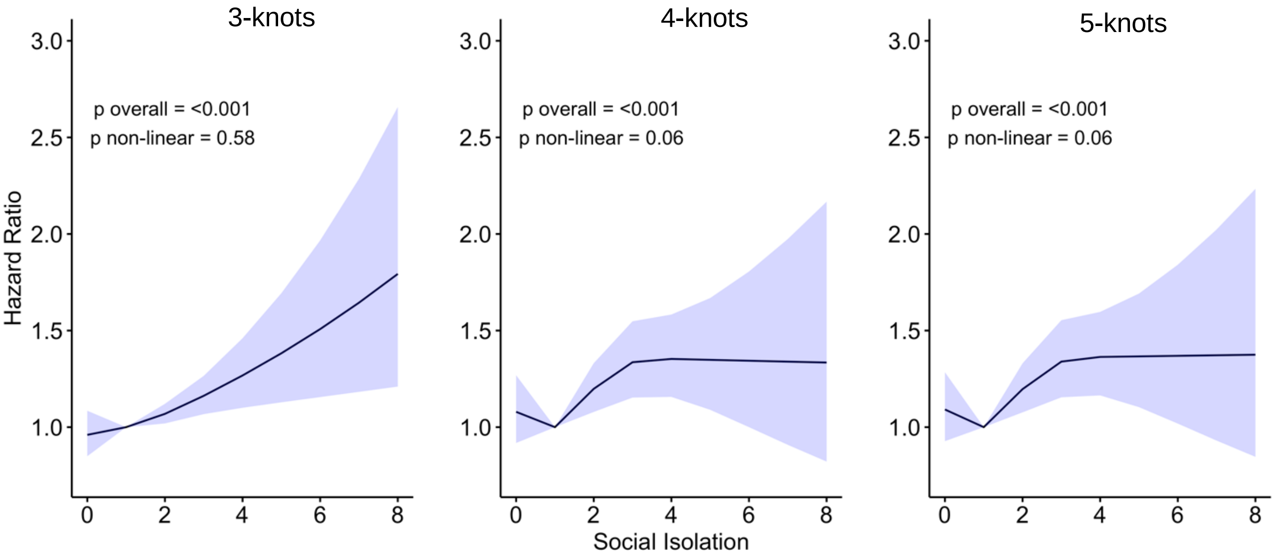

Panel B

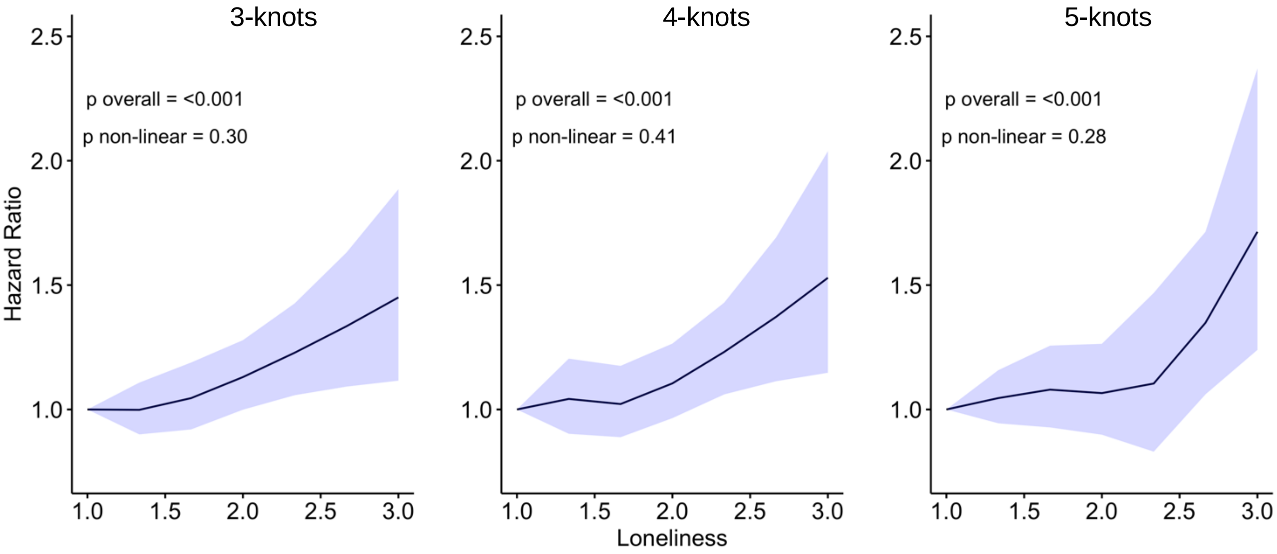

© 2022 Golaszewski NM et al. *JAMA Network Open*.
